# Evaluation of P450 monooxygenase activity in lyophilized recombinant *E. coli* cells compared to resting cells

**DOI:** 10.1186/s13568-021-01319-0

**Published:** 2021-12-04

**Authors:** Thomas Hilberath, Alessandra Raffaele, Leonie M. Windeln, Vlada B. Urlacher

**Affiliations:** 1 Institute of Biochemistry, Heinrich-Heine University Düsseldorf, Universitätsstraße 1, 40225 Düsseldorf, Germany; 2 Present Address: Department of Biotechnology, Delft University of Technology, van der Maasweg 9, 2629HZ Delft, The Netherlands; 3 Present Address: School of Chemistry, University of Southampton, B30, University Road, SO17 1BJ Southampton, UK

**Keywords:** Cytochrome P450, Whole-cell biotransformation, Lyophilized cells, Cofactor regeneration

## Abstract

Cytochromes P450 catalyze oxidation of chemically diverse compounds and thus offer great potential for biocatalysis. Due to the complexity of these enzymes, their dependency of nicotinamide cofactors and redox partner proteins, recombinant microbial whole cells appear most appropriate for effective P450-mediated biocatalysis. However, some drawbacks exist that require individual solutions also when P450 whole-cell catalysts are used. Herein, we compared wet resting cells and lyophilized cells of recombinant *E. coli* regarding P450-catalyzed oxidation and found out that lyophilized cells are well-appropriate as P450-biocatalysts. *E. coli* harboring CYP105D from *Streptomyces platensis* DSM 40041 was used as model enzyme and testosterone as model substrate. Conversion was first enhanced by optimized handling of resting cells. Co-expression of the alcohol dehydrogenase from *Rhodococcus erythropolis* for cofactor regeneration did not affect P450 activity of wet resting cells (46% conversion) but was crucial to obtain sufficient P450 activity with lyophilized cells reaching a conversion of 72% under the same conditions. The use of recombinant lyophilized *E. coli* cells for P450 mediated oxidations is a promising starting point towards broader application of these enzymes.

**Supplementary Information:**

The online version contains supplementary material available at 10.1186/s13568-021-01319-0.

## Introduction

Cytochromes P450 (CYPs or P450s) are versatile heme-containing enzymes that catalyze oxidation reactions in the presence of molecular oxygen and NAD(P)H. Due to their ability to introduce one atom of molecular oxygen into a vast variety of organic molecules under mild reaction conditions with often high chemo- and regioselectivity, these enzymes have been recognized as attractive targets with high potential for biotechnological applications (Bernhardt 2006; Girvan and Munro 2016; Kelly and Kelly 2013; Lundemo and Woodley 2015). Generally, whole-cell biocatalysis seems appealing because it allows avoiding cell lysis and enzyme isolation (Wachtmeister and Rother 2016). Enzymes are protected by the cell environment from the harmful influence of reaction components (Schrewe et al. 2013; Willrodt et al. 2015). In case of NADH and NADPH dependent enzymes like P450s, these cofactors can be continuously regenerated via metabolism of the host cell, or optionally by the use of heterologous cofactor regenerating enzymes and co-substrates (Hanlon et al. 2007). With regard to P450 enzymes, whole-cell biocatalysis might be particularly attractive because electrons from NAD(P)H are transferred via one or two redox partner proteins to the catalytically active heme. Co-expression of the enzymes belonging to a P450 redox chain in one microbial cell appears more attractive than separate expression and isolation of several enzymes.

Despite the apparent advantages of whole-cell systems for P450-catalyzed reactions, their application is often associated with challenges like substrate/product toxicity for the microbial cell and limited substrate and product transfer across the cell membrane (Bernhardt and Urlacher 2014; Lundemo and Woodley 2015). Whereas substrate toxicity can be overcome by using more stable hosts, improved substrate uptake can be achieved by co-expression of transporter proteins (Karande et al. 2018; Mi et al. 2014; Tieves et al. 2016), cell permeabilization (Janocha and Bernhardt 2013) or other commonly used procedures like freezing and thawing (Bracco et al. 2013; Lundemo et al. 2016). In case of hydrophobic substrates of P450 enzymes, their low solubility in aqueous solution represents an additional drawback for biocatalysis. To increase substrate solubility organic solvents are often added, which might negatively affect the whole-cell biocatalysts either. To this end, usage of lyophilized recombinant microbial cells carrying the target enzymes has been reported as an attractive alternative to both, microbial cells and isolated enzymes, because they allow working at high organic solvent concentrations and do not face the problem of substrate transport through the membrane (Jakoblinnert and Rother 2014).

**Fig. 1:**
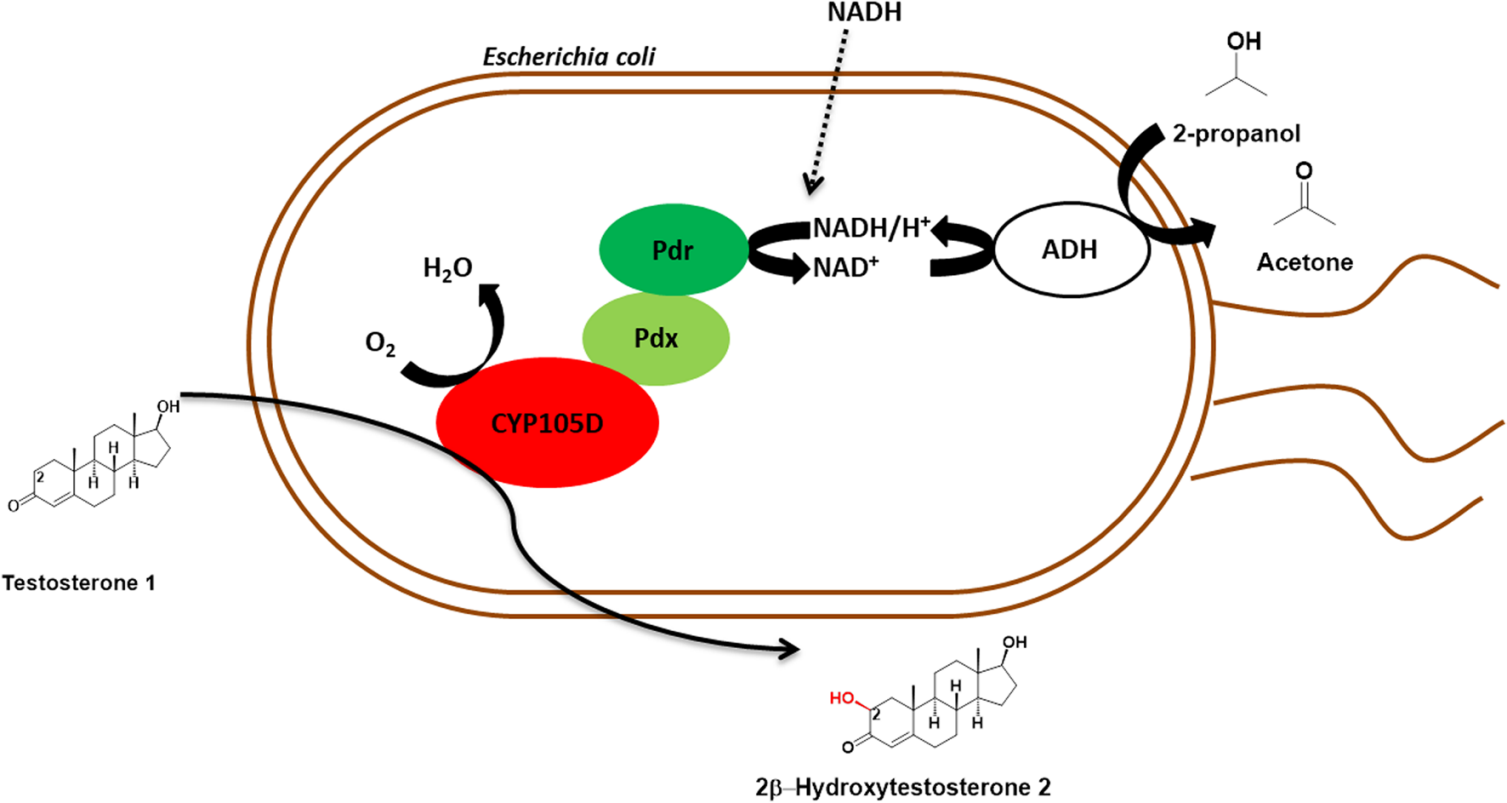
Schematic overview of the whole-cell biocatalyst expressing CYP105D from *S. platensis* for the oxidation of testosterone **1**. Putidaredoxin reductase (Pdr) and putidaredoxin (Pdx) from *P. putida* are used as redox partners for CYP105D. Alcohol dehydrogenase (ADH) from *R. erythropolis* was implemented for cofactor regeneration using propan-2-ol as sacrificial substrate and solvent for testosterone solubilization

In this respect, it is important to explore the use of lyophilized recombinant *E. coli* cells for the P450-mediated biocatalysis and compare them with the better investigated whole-cell preparations. In this work we used as model system the recently characterized CYP105D from *Streptomyces platensis* DSM 40041 that accepts a broad range of substrates including testosterone **1** (Hilberath et al. 2020). Oxyfunctionalized steroids like 2β-hydroxytestosterone **2** are of high pharmaceutical interest as drug precursors and human drug metabolites (Kiss et al. 2015). Testosterone **1** is a common steroid substrate often applied to evaluate the activity of P450s of prokaryotic and eukaryotic origin (Agematu et al. 2006; Geier et al. 2013; Kille et al. 2011; Zehentgruber et al. 2010). We chose this substrate for this study due to its low solubility in water and relatively large size which impair substrate uptake by recombinant *E. coli* cells. An *E. coli* C43 (DE3) whole-cell biocatalyst coexpressing CYP105D with the NADH-dependent putidaredoxin reductase (Pdr) and putidaredoxin (Pdx) on two plasmids was constructed and used for oxidation of testosterone **1** to 2β-hydroxytestosterone **2** (Fig. 1). Different whole-cell handling procedures in combination with membrane permeabilizing and solubilizing agents were compared to address the substrate transport issue. The implementation of an alcohol dehydrogenase for cofactor regeneration in recombinant *E. coli* allowed us to use recombinant lyophilized *E. coli* cells for the P450-mediated oxidation of testosterone **1 **and paved the way for an easy-to-use whole-cell system of P450 enzymes.

## Materials and methods

### Chemicals and strains

*E. coli* DH5α was used for cloning (Clontech) while *E. coli* OverExpress C43(DE3) (Lucigen) was applied for heterologous expression. Catalase from *bovine*, testosterone **1**, (2hydroxypropyl)-β-cyclodextrin and polymyxin B sulphate were obtained from Sigma Aldrich. Other chemicals were of analytical grade and purchased from commercial sources.

### Cloning and gene expression

The gene *cyp105D* from *S. platensis* (GenBank accession no. OSY47991) was cloned using conventional cloning methods in the expression vector pET22b between the recognition sites for the endonucleases *Nde*I and *Xho*I resulting in pET22b-*cyp105D*. Gibson assembly was used to clone the genes coding for alcohol dehydrogenase (*re-adh*, GenBank accession no. CAF04319), putidaredoxin reductase (*camA*, GenBank accession no. BAA00413) and putidaredoxin (*camB*, GenBank accession no. BAA00414) in the pCOLA-Duet vector (Gibson et al. 2009) resulting either in pCOLADuet-PP or pCOLADuet-PP-RE, respectively. Details on primer sequences and vector properties are provided in the Supplementary information (Tables S1 and S2).

The genes encoding for CYP105D and redox partners were expressed from a two-plasmid system in *E. coli* C43 (DE3) similar as described previously (Worsch et al. 2018). For gene expression, 100 mL TB-medium was inoculated with an overnight culture of the respective recombinant *E. coli* strain to an OD_600_ of 0.05. The cultures were grown in 1 L flasks at 37 °C and 180 rpm for 2.5–3 h. At an OD_600_ of ≈1.0, 500 µM 5-aminolevulinic acid was added and expression of target genes was induced with 500 µM isopropyl β-d-1-thiogalactopyranoside (IPTG). All cultures were incubated at 20 °C and 140 rpm for 20 h after induction.

### **Preparation of recombinant*E. coli*cells**

Resting *E. coli* C43 (DE3) cells, carrying pET22b-*cyp105D* and pCOLADuet-PP (if not stated otherwise), were investigated. After cultivation, the culture broth was split to several 50 mL falcon tubes and cells were harvested by centrifugation for at least 20 min at 5250 *g* and 4 °C. Cell pellets were then washed with 25 mL Phosphate Sucrose EDTA (PSE)-buffer (6.75 g/L KH_2_PO_4_, 85.5 g/L sucrose, 0.93 g/L EDTA-Na_2_*2 H_2_O, pH 7.5). EDTA was added to destabilize the outer membrane by chelating metal ions. Sucrose was added as stabilizing agent during cell freezing. The cells were treated in different ways as described in the Results section. Prior to the whole-cell biotransformation, cell preparations except for the lyophilized cells were adjusted to a cell wet weight (cww) of 100 mg/mL.

Lyophilized cells were obtained using a Christ alpha 2–4 LSCplus (Martin Christ Gefriertrocknungsanlagen GmbH, Germany). For that purpose, cells were spread in a crystallization bowl and frozen at − 80 °C. The cryoprotectant glycerol was added at a final concentration of 5% (w/v) (Additional file 1: Fig. S1). Lyophilization was conducted for at least one day at − 80 °C under vacuum. Lyophilized cells were then transferred to a 50 mL reaction tube and stored at − 20 °C.

### Preparation of crude cell extracts

Before cell disruption, cells were resuspended in 5 mL cold PSE-buffer supplemented with 0.1 mM phenylmethylsulfonyl fluoride (PMSF). The cell suspension was disrupted by sonication on ice (Branson Ultrasonics Sonifier 250; 3 × 1.5 min, 4% amplitude, duty cycle 4). Between the cycles the cell suspension was incubated for 2 min on ice. Cell debris was removed by centrifugation (40.000 *g*, 25 min and 4 °C). The soluble fraction (crude cell extract) was collected and directly used for determination of the P450-concentration and the ADH-activity. For cell dry weight (cdw) determination, 200–250 µL of the wet cell suspension were transferred to a dry 1.5 mL reaction tube. After centrifugation for 2 min at 13.500 *g* at room temperature, the supernatant was discarded and the cell pellets dried for 48 h at 60 °C before weighing. All measurements were performed in triplicates.

### Whole-cell biocatalysis

Biotransformations were performed in 2 mL Eppendorf tubes with 500 µL PSE-buffer containing resting cells in a final concentration of 50 mg/mL (cww) or lyophilized cells in a final concentration of 10 mg/mL (cdw), 1 × nutrient solution (6 mM glucose, 6 mM lactose and 12 mM citrate in PSE-buffer) and 1 mM testosterone **1** (in 5% (*v/v*) cosolvent final concentration). The tested co-solvents were propan-2-ol and acetone. Optionally, 10–100 µg/mL polymyxin B or 1–10 mM (2-hydroxypropyl)-β-cyclodextrin were added. 2 mL reaction tubes with open lids were incubated at 25 °C up to 20 h at 1100 rpm in an Eppendorf shaker. At different time points 50-200 µL aliquots were taken for extraction with 1 mL ethyl acetate. 200 µM progesterone was added as internal standard. After phase separation the organic phase was transferred to a new reaction tube and concentrated under reduced pressure. The analytes were resolved in methanol for LC/MS analysis. Conversions were calculated from the sum of detected product peak areas relative to the substrate peak area either via PDA- or MS-analysis. The ratio of 2β-hydroxytestosterone was calculated from the sum of all peak areas in the MS-or PDA-chromatograms.

#### Determination of P450-concentration and ADH-activity

Concentrations of P450 in crude cell extracts were calculated based on CO-difference spectra using the extinction coefficient ε_450_ = 91 mM^−1^ cm^−1^ as published elsewhere (Omura and Sato 1964). 2 × 950 µL of protein sample, diluted in PSE-buffer if necessary, were filled into plastic cuvettes and placed in a double-beam photometer (Perkin Elmer). After blanking, one of the samples was exposed to CO for a few seconds. Next, 50 µL of a 1 M sodium dithionite stock solution was added and a difference spectrum between 400 and 500 nm recorded. The measurements were continued until a constant absorption maximum was reached.

ADH-activity was measured in a continuous photometric assay monitoring NADH formation at 340 nm (ε_340_ = 6.22 mM^−1^ cm^−1^) in presence of propan-2-ol as substrate. Reaction mixtures contained 50 mM Tris-HCl with 10 mM MgCl_2_ (pH 8), 649 mM propan-2-ol 5% (v/v)) and 50 µL of crude cell extract in appropriate dilution. After incubation for 2 min at 25 °C, the reaction was started by adding 0.5 mM NAD^+^. The increase of absorption caused by NADH formation was tracked for 120 s at 25 °C in a double-beam photometer (Perkin Elmer). The initial slope (ΔA_340_/min) between 20 and 80 s was linear and thus used to calculate the activity [U/g_CDW_]. 1 U is defined as the amount of enzyme which is needed to convert 1 µmol substrate in 1 min under assay conditions. All measurements were done in duplicates.

#### Product analysis

Product analysis was conducted by liquid chromatography coupled to mass spectrometry (LC/MS) on a Prominence/LCMS 2020 device (Shimadzu). Analytes were separated with a flow rate of 1 mL/min at 30 °C on a Chromolith® Performance RP18e column (100 × 4.6 mm, Merck) using methanol as solvent B and ddH_2_O with 0.1 % formic acid as solvent A. 1 µL of each sample was injected. The substances were ionized by electron spray ionization (ESI) and atmospheric pressure chemical ionization (APCI) in a dual ionization mode. Masses were detected in positive scan mode in a range between 100 and 500 *m/z*. Additionally, photo diode array (PDA) chromatograms at 254 nm were recorded. The conditions for chromatographic separation were carried out as described previously (Hilberath et al. 2020). An overview of all products formed during P450-mediated oxidation of testosterone **1** is provided in Additional file 1: Table S3. Exemplary MS-chromatograms are provided in Additional file 1: Fig. S2.

## Results

### Construction of the whole-cell system with CYP105D and redox partners

**Fig. 2:**
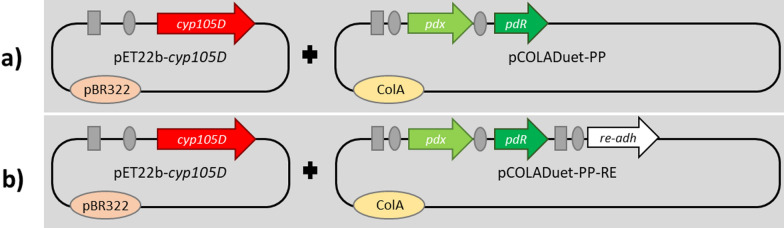
Plasmid combinations used in this study for whole-cell biocatalyst design. The P450 gene *cyp105D* is always encoded on the pET22b-vector. Redox partner genes (*pdx/pdr*) are integrated in MCSI of pCOLADuet. The *re-adh* gene (**b**) is integrated in the MCSII of the pCOLADuet-vector. Gray squares represent the T7-promoter; gray circles indicate the ribosome binding site

The gene coding for CYP105D from *S. platensis* was co-expressed with a redox partner system consisting of the NADH-dependent putidaredoxin reductase (Pdr) and putidaredoxin (Pdx) from two plasmids (Fig. 2A). The gene *cyp105D* was cloned in the pET22b-vector, whereas *pdr* and *pdx* were integrated in the first multiple cloning site of the pCOLADuet-vector. The resulting expression vectors pET22b-*cyp105D* and pCOLADuet-PP were both used for transformation of *E. coli* C43 (DE3). The expression of *cyp105D*, *pdr* and *pdx* in *E. coli* C43 (DE3) was tracked by SDS-PAGE (Additional file 1: Fig. S3) and indicated accumulation of the P450. Soluble production of CYP105D was confirmed by measuring a P450-concentration of 278 ±11 nmol/g_CDW_ via CO-difference spectra.

### Comparison of different cell preparations

An overall challenge of whole-cell biocatalysis is the transfer of hydrophobic substrates and products across the cell membrane (Chen 2007). Since testosterone **1** is a large compound with low solubility in water, we suspected difficulties in substrate intake by the cell. Different straight-forward methods for physical cell treatments such as freeze and thawing, sonication, or lyophilization can be used to improve substrate transfer and achieve effective P450 whole-cell biocatalysis. To systematically investigate and compare these methods and to identify the optimal cell preparation for sufficient transport of testosterone **1** through the membrane, the following *E. coli* cell preparations were used in this study:

(i) Resting cells obtained directly after cultivation and centrifugation without further treatment (‘non frozen’).
(ii) Resting cells obtained directly after cultivation and centrifugation and three sonication cycles after resuspension in PSE-buffer without any additional freezing step (‘sonified’).
(iii) Resting cells which were frozen at − 20 °C as cell pellet for at least 24 h (‘frozen cell pellet’).
(iv) Resting cells which were frozen at − 20 °C as cell suspension in PSE-buffer at a concentration of 100 mg/mL (‘frozen cell suspension’).
(v) Lyophilized cells obtained from resting cells which were frozen in a crystallization bowl directly after cultivation and centrifugation (‘lyophilized cells’).

**Fig. 3:**
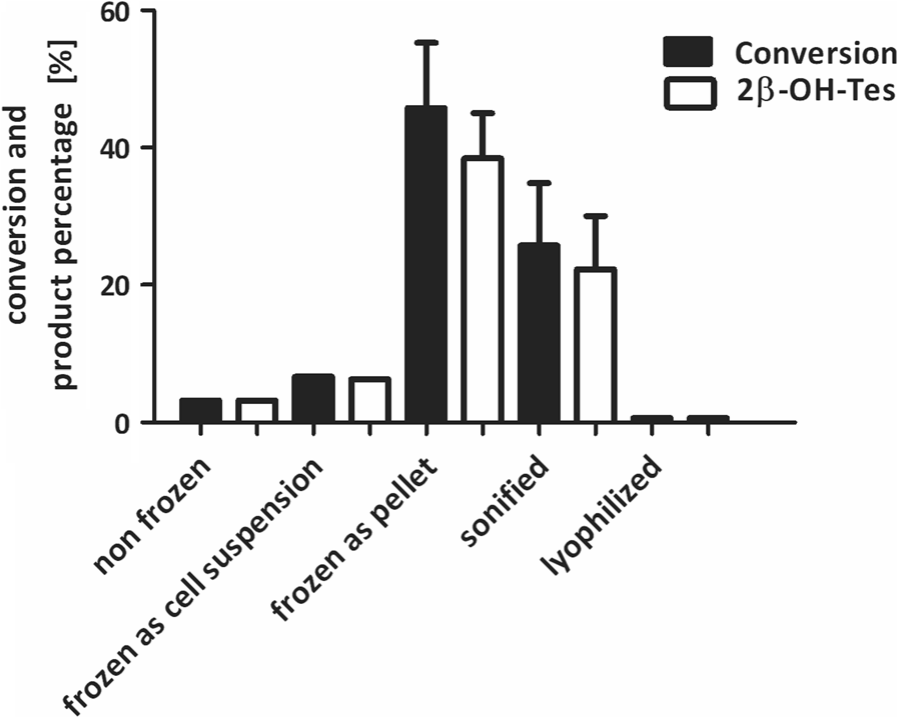
Effect of different handling of resting cells of *E. coli* C43 (DE3) pET22b-*cyp105D* + pCOLADuet-*pdx*-*pdr* on testosterone **1** conversion and formation of 2β-hydroxytestosterone **2** (2β-OH-Tes). Reaction conditions: 50 mg/mL wet cells in 0.5 mL Phosphate Sucrose EDTA (PSE)- buffer with 1 x nutrient solution, pH 7.5 in 2 mL reaction tubes; 1 mM testosterone **1** dissolved in 5% (v/v) propan-2-ol as final concentration, 25 °C, 1100 min^−1^ shaking frequency, reaction time 20 h. All measurements were performed in technical duplicates. In case a standard deviation is given, experiments were additionally conducted in biological duplicates

The lowest conversion of 3% was observed with resting cells used immediately after cultivation and centrifugation (‘non frozen’) (Fig. 3). Freeze-thawing of *E. coli* cells has been reported to destabilize cell membrane by releasing some cell components, which make it more permeable. Indeed, freezing the cells at − 20 °C with subsequent thawing had a beneficial effect on conversion (Fig. 3). However, it did matter in which manner the cells were frozen. If cells were first resuspended in buffer and then frozen at -20 °C (‘frozen as cell suspension’), the conversion was lower as compared to the procedure when the cell paste after centrifugation was frozen, thawed and resuspended in buffer just before the biotransformation (‘frozen as pellet’) (6% vs. 46%). The conversion achieved with the best performing resting cells frozen at − 20 °C (‘frozen as pellet’) was around 1.8-fold higher in comparison to cells which were treated by sonication without any freezing step (‘sonified’) with which the conversion was about 26%.

### Effect of solubilizing and membrane permeabilizing agents

Apart from physical methods, substrate solubilizing and membrane permeabilizing agents were reported to improve conversion by P450 whole-cell biocatalysts (Bracco et al. 2013; Janocha and Bernhardt 2013; Tieves et al. 2016). 

**Fig. 4:**
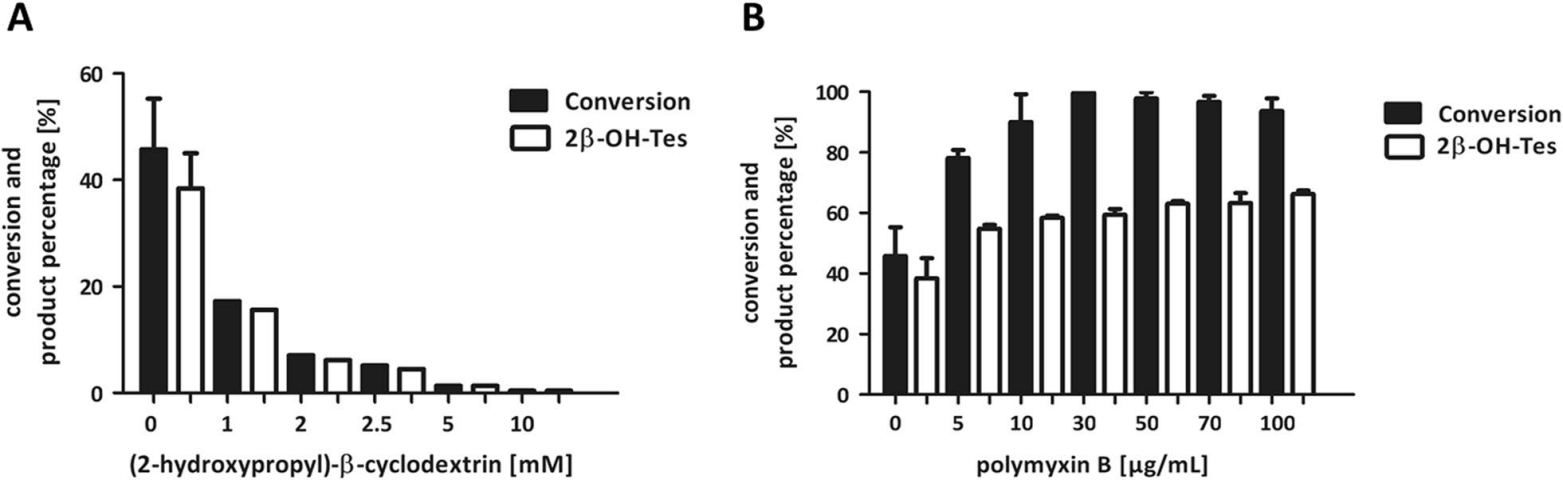
Conversion of testosterone **1** and formation of 2β-hydroxytestosterone 2 (2β-OH-Tes) by *E. coli* C43 (DE3) pET22b-*cyp105D* + pCOLADuet- *pdx*-*pdr* in presence of different concentrations of (2-hydroxypropyl)-β-cyclodextrin (**A**) or polymyxin B (**B**). Reaction conditions: 50 mg/mL wet cells in 0.5 mL Phosphate Sucrose EDTA (PSE)-buffer with 1 × nutrient solution, pH 7.5 in 2 mL reaction tubes; 1 mM testosterone **1** dissolved in 5% (v/v) propan-2-ol as final concentration; 25 °C, 1100 min^−1^ shaking frequency, reaction time 20 h. Cells were frozen at − 20 °C for preparation of ‘frozen cells.’ (2-hydroxypropyl)-β-cyclodextrin or polymyxin B was additionally added to the best performing wet cell biocatalyst (‘frozen as cell pellet’). All measurements were performed in technical duplicates. In case a standard deviation is given, experiments were additionally conducted in biological duplicates

Thus, after identification of the most suitable whole-cell preparation (‘frozen as pellet’), we aimed to increase conversion further by addition of cyclodextrins (Fig. 4A) and the membrane-permeabilizing peptide polymyxin B (Fig. 4B). Cyclodextrins are solubilizing agents that possess a hydrophilic outer surface and a hydrophobic cavity in which they can accommodate hydrophobic molecules in aqueous solution (Loftsson and Brewster 1996; Rühlmann et al. 2017). For whole-cell conversions of steroids (2-hydroxypropyl)-β-cyclodextrin was frequently used (Bracco et al. 2013; Fokina et al. 1997). In the present case, the addition of (2-hydroxypropyl)-β-cyclodextrin had a negative effect on conversion. In comparison to the whole-cell conversion without cyclodextrins, the equimolar addition of 1 mM (2-hydroxypropyl)-β-cyclodextrin already led to an approximately 3-fold decrease of substrate conversion (17%). Increasing cyclodextrin concentrations caused a further decrease in conversion.

Other than (2-hydroxypropyl)-β-cyclodextrin, addition of polymyxin B had a positive effect on conversion. Polymyxin B is a peptide antibiotic that permeabilizes the outer membrane of *E. coli* (Lounatmaa and Nanninga 1976). When using chemical permeabilization methods, it is important to test different concentrations of the respective reagents, as too high a concentration of the reagent can have a negative effect on the activity of the whole-cell biocatalyst, leading to cell lysis in the worst case (Chen 2007; Fontanille and Larroche 2003). In our study, addition of 5 µg/mL polymyxin B resulted in a improved substrate conversion of 78%. Higher polymyxin B concentration increased substrate conversion until almost 100%. At high polymyxin B concentration of 100 µg/ml the reaction mixture turned reddish, which indeed can indicate cell lysis.

### Effect of cofactor regeneration

Activity of the lyophilized P450 whole-cell biocatalyst was less than 1% (Fig. 3). We assumed that loss of activity in lyophilized cells was attributed to insufficient cofactor supplementation. This bottleneck has already been addressed for P450 based whole-cell systems where co-expression of NAD(P)H regenerating enzymes such as glucose dehydrogenase or glycerol dehydrogenase can help to increase P450 activity (Schewe et al. 2008; White et al. 2017). However, less is known about the effect of co-expression of NAD(P)H regenerating enzymes in lyophilized cells.

**Fig. 5:**
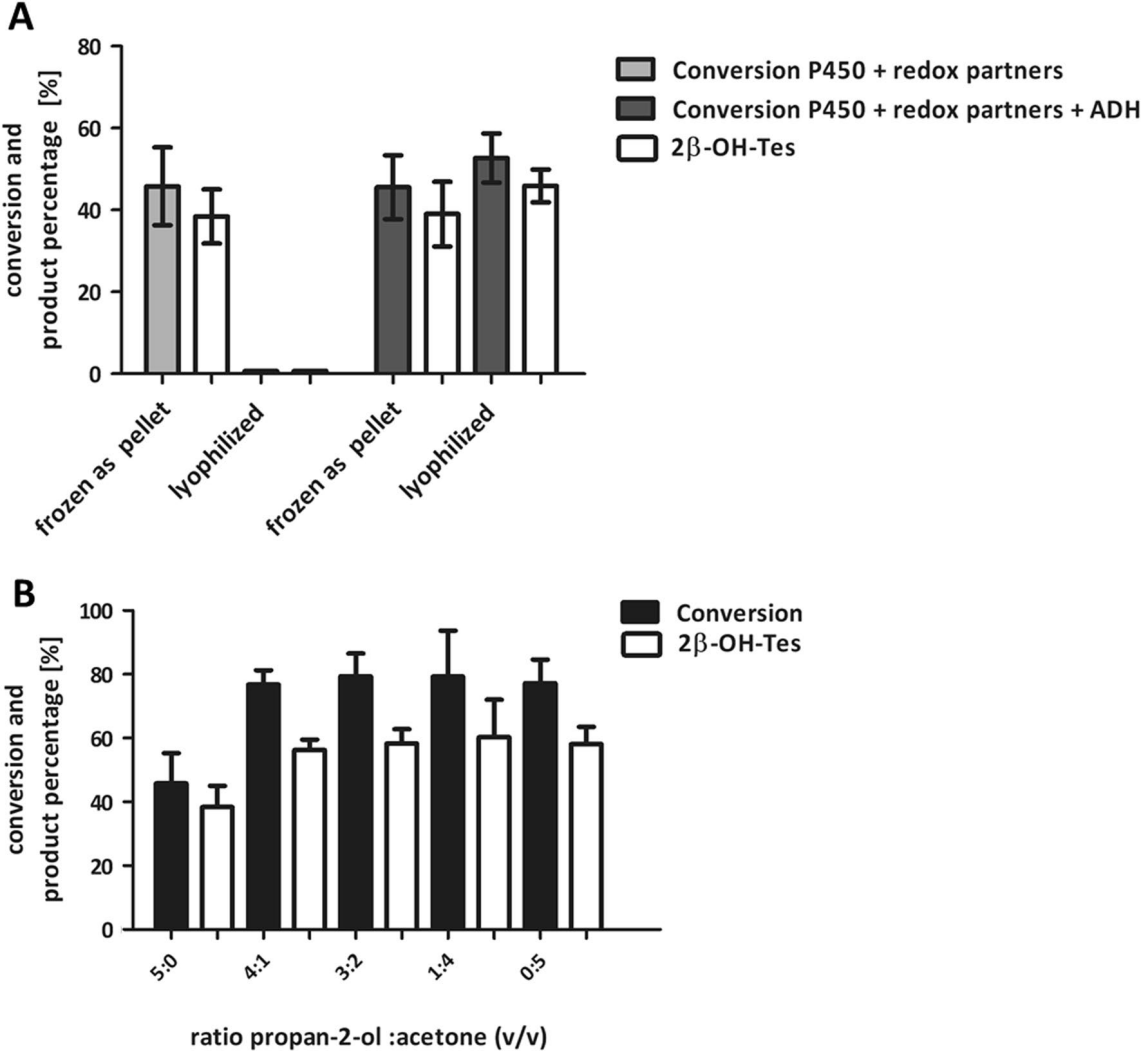
**A** Influence of cofactor regeneration by Re-ADH in *E. coli* C43 (DE3) pET22b-*cyp105D* + pCOLADuet- *pdx*-*pdr-adh* on testosterone** 1** conversion and formation of 2β-hydroxytestosterone **2** (2β-OH-Tes). **B** Effect of different ratios of propan-2-ol and acetone on testosterone 1 conversion and formation of 2β-OH-Tes mediated by the wet cells without ADH (P450 + redox partners). The best performing wet cell biocatalyst (`frozen as cell pellet’) was investigated (see Fig. 3). Reaction conditions: 10 mg/mL lyophilized cells or 50 mg/mL wet cells in 0.5 mL Phosphate Sucrose EDTA (PSE)-buffer with 1 × nutrient solution, pH 7.5 in 2 mL reaction tubes; 1 mM testosterone 1 dissolved in 5% co-solvent (v/v) final concentration, 25 °C, 1100 min^−1^ shaking frequency, reaction time 20 h. All measurements were performed in technical duplicates. In case a standard deviation is given, experiments were additionally conducted in biological duplicates

As it would be advantageous to use lyophilized cells due to their easy handling, we further investigated if cofactor supply affected their catalytic performance in this case. To ensure cofactor regeneration in lyophilized cells, we additionally cloned the gene encoding for the alcohol dehydrogenase from *Rhodococcus erythropolis* DSM 43297 in the plasmid downstream of *pdx* and *pdr* (Fig. 2B). The NAD^+^-dependent alcohol dehydrogenase from *R. erythropolis* DSM 43297 (Re-ADH) (Abokitse and Hummel 2003) catalyzes oxidation of the cheap sacrificial substrate propan-2-ol to acetone thereby reducing NAD^+^ to NADH (Kroutil et al. 2004). Hence, we used propan-2-ol as substrate of Re-ADH and simultaneously as co-solvent to dissolve testosterone **1**. The P450 concentration in the cell was marginally affected by co-expression of an additional enzyme (278 ±11 nmol/g_CDW_ vs. 268 ±2 nmol/g_CDW_) as determined from CO-difference spectra. NADH production during propan-2-ol oxidation was evaluated by a photometric assay and was only detected with *E. coli* cells expressing Re-ADH (52 ± 0 U/g_CDW_) and not with another strain, which indicated that this ADH was successfully expressed. The co-expression of Re-ADH had no effect on the activity of the best-performing resting wet cells (‘frozen as cell pellet’) (46% conversion). However, a particularly advantageous effect on activity was observed for the lyophilized whole-cell biocatalyst showing a higher conversion of 53% (Fig. 5A). This effect indicates that targeted cofactor regeneration is crucial to support P450 activity in lyophilized cells.

In order to further validate this hypothesis, we investigated the influence of external NADH on the activity of lyophilized cells since NADH might get lost or degraded during lyophilization (Zehentgruber et al. 2010). NADH was added to lyophilized cells in different concentrations and at different time points up to four times during biotransformation (Aditional file 1: Fig. S4). The activity of the lyophilized cells without Re-ADH could be increased only very slightly by NADH-addition (0.25–1 mM final concentration) independent of the time point and amount of added NADH (max conversion 3%). However, the supplementation of 0.5 mM NADH after 4 h to the lyophilized cells where Re-ADH was present resulted in a 1.4-fold increase in activity towards testosterone **1**. Testosterone **1** conversion of 72% with lyophilized cells was similar or even slightly higher than that observed with resting cells (Table 1).

**Table 1:** Effect of external NADH addition on the activity of lyophilized P450 whole-cell catalysts

Lyophilized *E.coli* C43 (DE3) harboring	Testosterone conversion [%]		Formation of 2βhydroxytestosterone [%]	
	− NADH	+ NADH	− NADH	+ NADH
pET22b-*cyp105D* + pCOLADuet-*pdx*-*pdr*	≤ 1	3	≤ 1	3
pET22b-*cyp105D* + pCOLADuet-*pdx*-*pdr*-*adh*	53 ± 6	72 ± 5	46 ± 4	61± 4

Reaction conditions: 10 mg/mL lyophilized cells in 0.5 mL Phosphate Sucrose EDTA (PSE)-buffer with 1 × nutrient solution, pH 7.5, in 2 mL reaction tubes, 1 mM testosterone **1** dissolved in 5% (v/v) propan-2-ol final concentration, 25 °C, 1100 rpm, 20 h reaction time. 0.25 mM NADH was added up to four times at 0 h, 2 h, 4 and 6 h incubation. For the cells co-expressing the *adh*, 0.5 mM NADH were added after 4 h. Experiments were performed in technical duplicates

Acetone is formed during NADH formation by Re-ADH and thus may contribute to change in solubility and cellular uptake of the substrate testosterone **1** (Kroutil et al. 2004). Furthermore, acetone is a common organic solvent used for the permeabilization of cell membrane (Kiss et al. 2015; Lundemo et al. 2016). The oxidation of propan-2-ol to acetone is a reversible reaction, which leads to a thermodynamic equilibrium and consequently to different ratios of the two co-solvents over time (Schroer et al. 2007). We analyzed substrate conversion catalyzed by wet cells and lyophilized cells, both containing the P450 system but no Re-ADH, by testing different ratios of the co-solvents propan-2-ol and acetone (Fig. 5B). Increasing acetone concentrations had a positive effect on conversion by the cells without Re-ADH and resulted in a 1.5-fold increase of up to 79% conversion. However, this effect was only observed when wet cells were used. When lyophilized cells were applied, conversion with increasing acetone concentrations was still less than 1% (data not shown).

## Discussion

Lyophilized recombinant *E. coli* cells are attractive for practical use, because they allow easier preparation and storage compared to wet resting cells. Furthermore, lyophilized cells can be used in reaction systems with higher amount of organic co-solvents, allowing higher concentrations of hydrophobic substrates (Wachtmeister et al. 2014). This is particularly interesting because typical substrates of P450s are hydrophobic. A CYP105D-based *E. coli* whole-cell biocatalyst was constructed with the aim of establishing a procedure that is based on the use of lyophilized cells. The hydroxylation of testosterone **1** to 2β-hydroxytestosterone **2** was chosen as model reaction.

First, we investigated the effect of cell membrane disruption on substrate conversion. To this end, the effect of different cell handling procedures as well as chemical permeabilization methods on substrate conversion were analyzed. The highest conversion was obtained when cells were frozen as cell paste rather than as cell suspension (Fig. 3). As previously reported, slow freeze-thawing mainly released components of the outer membrane, whereas fast freeze-thawing caused a more drastic decay, also releasing cytoplasmic components (Souzu 1980). In our experiments, individual cells resuspended in buffer can be frozen and thawed faster than cell paste. Additionally, ice crystals might also have an impact on the release of cell components. On this basis, we hypothesize that cells resuspended in buffer lose cytoplasmic components after freeze-thawing and are thus less stable and active. Other cell treatments such as sonication also resulted in lower conversion. Sonication is considered an efficient method for cell disintegration and is usually used for the isolation of intracellular proteins from *E. coli* (Feliu et al. 1998). Controlled sonication may allow partial cell disintegration and thus improve substrate intake. In our experiments testosterone **1** conversion with sonified cells was indeed higher than with non-frozen cells or with cell frozen as suspension but lower that that achieved with cells frozen as pellet. We suggest, that in the sonified cells, P450 and redox partner proteins become better accessible for the substrate but are less stable than in frozen resting cells or are partially destabilized by increased temperatures developed during sonication.

Apart from the different physical cell treatments, we investigated the effect of (2-hydroxypropyl)-β-cyclodextrin and polymyxin B (Fig. 4). Cyclodextrins build host-guest complexes with hydrophobic substances and enhance their solubility and simultaneously reduce their possible toxic effects (Singh et al., 2002). In our previous work, the addition of methyl- β-cyclodextrin to recombinant *E. coli* and *P. putida* resting cells had a positive effect on *n-*octane hydroxylation with a P450, although the effect on *E. coli* was weaker (Tieves et al. 2016). In this work, addition of (2-hydroxypropyl)-β-cyclodextrin did not improve but reduced the conversion with the frozen cells (Fig. 4A). Probably, (2-hydroxypropyl)-β-cyclodextrin did not increase the solubility of 1 mM testosterone **1** over propan-2-ol. On the other hand, it is known that with increasing cyclodextrin concentrations lower amounts of free substrate and/or product in solution are present (Kiss et al. 2015; Singer et al. 1991). In the present case, it is assumed that the substrate got trapped by the cyclodextrin and thus is not accessible for the whole-cell biocatalyst any longer. However, the exact reason remains elusive and needs to be investigated further.

In contrary, almost complete conversion was achieved when the recombinant whole-cell biocatalyst was treated with polymyxin B (Fig. 4B). Addition of the cell permeabilizer polymyxin B has been reported to improve conversion of hydrophobic substrates by recombinant *E. coli* as P450 whole-cell biocatalysts (Janocha and Bernhardt 2013; White et al. 2017). Consequently, this cell permeabilizing agent seems well-suited for P450 whole-cell catalysis, both in relation to the aforementioned studies and in comparison to the here investigated lyophilized cells. However, high polymyxin B concentrations can lead to cell lysis, which we supposed to happen at 100 µg/ml. Furthermore, depending on the toxicity and concentration of the substrates and products, various effects of polymyxin B on *E. coli* whole-cell biocatalysts have been described. While Janocha et al. found a positive effect of polymyxin B for the biotransformation of abietic acid, a negative effect on the P450 whole-cell catalyst was observed by White et al. for hydroxylation of *n*-octane in the whole-cell system, which the authors attributed to the too rapid accumulation of the toxic product 1-octanol (Janocha and Bernhardt 2013; White et al. 2017). To this end, a general use of polymyxin B for P450 whole cell catalysis is difficult (White et al. 2017). Additionally, the use of the antibiotic polymyxin B may be especially problematic for the production of pharmaceuticals with regard to antibiotic resistances and complete removing of this compound in downstream processing (Chokshi et al. 2019; Hapala 1997).

Likely, use of lyophilized cells as alternative is attractive because no additional compounds increase complexity of downstream processing or negatively affect activity of the whole-cell catalyst. Initially, the activity of lyophilized recombinant cells was very low (≤ 1% conversion) compared to the activity of wet resting cells (46% conversion). The lower activity of lyophilized cells could be attributed to insufficient cofactor regeneration. When Re-ADH was co-expressed to ensure cofactor regeneration, activities were comparable or even higher between lyophilized and wet cells (Fig. 5A). Under the optimal conditions, a conversion of 72% of 1 mM substrate was achieved. This activity is in the same range which was observed with isolated enzymes (Hilberath et al. 2020). The combination of P450s with heterologous redox partners for non-physiological substrates often results in high uncoupling which leads to unproductive NADH consumption (Bernhardt and Urlacher 2014). In the present case, the low conversion might reflect the uncoupling of the tested P450 system assuming that NADH cannot be regenerated by the metabolism in lyophilized *E. coli* cells. The increase in conversion catalyzed by the whole-cell biocatalyst with Re-ADH compared to the system without Re-ADH could be explained not only by the additional cofactor regeneration of ADH but also by the formation of acetone, which might have a positive effect on cell permeability (Fig. 5B). As this was observed only with wet and not with lyophilized cells, it supports the idea that targeted cofactor regeneration rather than improved substrate solubility and uptake is crucial to achieve P450 activity in lyophilized cells.

In conclusion, our results demonstrate that (i) handling procedure has a strong effect on the catalytic performance of recombinant P450-containing resting cells, (ii) lyophilized recombinant *E. coli* cells can be used for P450-mediated biocatalysis, when (iii) metabolism-independent regeneration of NAD(P)H is ensured. The use of these procedures illustrates interesting perspectives for convenient applications of cytochrome P450s for single- or multi-step reactions.

## Supplementary Information

**Additional file 1: Table S1**: Synthetic oligonucleotides for cloning. **Table S2**: Plasmids used in this study. **Table S3**: Analysis of testosterone **1** and metabolites **2**-**10** that were formed during CYP105D-mediated oxidation. **Figure S1**: Effect of freezing and glycerol addition during lyophilization on conversion catalyzed by *E. coli* C43 (DE3) pET22b-*cyp105D* + pCOLADuet- *pdx-pdr-adh*. *E. coli* cells were once or twice frozen at − 80°C and then lyophilized for either 24 h (black) or 48 h (grey). **Figure S2**: Exemplary LC/MS-chromatogram showing the oxidation of testosterone **1** to the products **2**-**10** by the CYP105D-based *E. coli* whole-cell biocatalyst (pink) in comparison to a negative control (black). **Figure S3**: SDS-PAGE analysis of *E. coli* C43 (DE3) strains for whole-cell biocatalysis. **Figure S4**: Effect of NADH addition on testosterone **1** conversion mediated by the lyophilized whole-cell catalyst without ADH. NADH was added up to four times (number in brackets) every 2 h.

## Data Availability

All data generated or analyzed during this study are included in this published article and its Additional files.

## Abbreviations

CYP: Cytochrome P450, Pdr:putidaredoxin reductase from *Pseudomonas putida*.
Pdx: Putidaredoxin from *Pseudomonas putida*.
Re-ADH: Alcohol dehydrogenase from *Rhodococcus erythropolis*.
NADH: Nicotinamide adenine dinucleotide.
