# Supplementary material for: Evaluation of P450 monooxygenase activity in lyophilized recombinant E. coli cells compared to resting cells
Source: AMB Express. 2021 Dec 4;11:162. doi: 10.1186/s13568-021-01319-0 (PMC8643389; doi:10.1186/s13568-021-01319-0)
Supplement: Supplementary file 1 — Additional file 1: Table S1: Synthetic oligonucleotides for cloning. Table S2: Plasmids used in this study. Table S3: Analysis of testosterone 1 and metabolites 2-10 that were formed during CYP105D-mediated oxidation. Figure S1: Effect of freezing and glycerol addition during lyophilization on conversion catalyzed by E. coli C43 (DE3) pET22b-cyp105D + pCOLADuet- pdx-pdr-adh. E. coli cells were once or twice frozen at − 80°C and then lyophilized for either 24 h (black) or 48 h (grey). Figure S2: Exemplary LC/MS-chromatogram showing the oxidation of testosterone 1 to the products 2-10 by the CYP105D-based E. coli whole-cell biocatalyst (pink) in comparison to a negative control (black). Figure S3: SDS-PAGE analysis of E. coli C43 (DE3) strains for whole-cell biocatalysis. Figure S4: Effect of NADH addition on testosterone 1 conversion mediated by the lyophilized whole-cell catalyst without ADH. NADH was added up to four times (number in brackets) every 2 h. [file 13568_2021_1319_MOESM1_ESM.pdf]

Evaluation of P450 monooxygenase activity in lyophilized recombinant *E. coli* cells compared with resting cells

Thomas Hilberath[1,2], Alessandra Raffaele[1], Leonie M. Windeln[1,3], Vlada B. Urlacher[1]*

[1]Institute of Biochemistry, Heinrich-Heine University Düsseldorf, Universitätsstraße 1, 40225 Düsseldorf, Germany

[2]Present address: Department of Biotechnology, Delft University of Technology, van der Maasweg 9, 2629HZ, Delft, The Netherlands

[3]Present address: School of Chemistry, University of Southampton B30, University Road, SO17 1BJ, Southampton, United Kingdom

* Corresponding author: Vlada B. Urlacher

Email: vlada.urlacher@uni-duesseldorf.de

Supplementary Material and methods

Table S1: Synthetic oligonucleotides for cloning. Restriction sites are underlined.

Primer name	DNA-sequence (5'-3')	Restriction enzymes	usage
F-READH-NdeI	CATCTTAGTATATTAGTTAAGTATAAGAAGGAGATATACATATGAAGGCAATCCAGTACACGAGAATC	*Nde*I/*Xho*I	Amplification of *re-adh*
RC-READH-XhoI	CTGGCGTTCAAATTTCGCAGCAGCGGTTTCTTTACCAGACTCGAGTTACAGACCAGGGACCACAACCG		
fw-PP-pCOLADuet	CAATTCCCCTGTAGAAATAATTTTGTTTAACTTTAATAAGGAGATATACCATGGCTTCTAAAGTAGTGTATGTGTCACATG	*Nco*I/*Bam*HI	Amplification of *camA* and *camB*
Rv-PP-pCOLADuet	CAAGCTTGTCGACCTGCAGGCGCGCCGAGCTCGAATTCGGATCCTCAGGCACTACTCAGTTCAGCTTTG		

Table S2: Plasmids used in this study.

vector (internal number)	genes with EMBL bank number	vector properties	reference/source
pET22b-*cyp105D* (THI 78)	*cyp105D* (EMBL-Bank: OSY47991)	SPL_00625 (*Nde*I,*Xho*I) cloned in pET22b	Hilberath et al. (2020)
pET-28a(+)-Re-ADH (FTI 94)	*re-adh* (EMBL-Bank: CAF04319)	*re-adh* Y174F cloned in pET28a	Abokitse and Hummel (2003)
pCOLADuet-PP (THI 87)	*camA* (EMBL-Bank: BAA00413) and *camB* (EMBL-Bank: BAA00414)	*camA* and *camB* (MCSI: *Nco*I, *Bam*HI) cloned in pCOLADuet1	Hilberath et al. (2020)
pCOLADuet-PP-RE (THI 103)	*camA* and *camB/re-adh*	*camA* and *camB* (MCSI: *Nco*I, *Bam*HI); *re-adh* (MCSII: *Nde*I, *Xho*I) cloned in pCOLADuet1	this work

Table S3: Analysis of testosterone 1 and metabolites 2-10 that were formed during CYP105D-mediated oxidation. Product identification and product distribution *in vitro* was described previously (Hilberath et al., 2020). Minor changes in retention time compared to the previous study are due to maintenance of the LC/MS device.

**Compound**	**Retention time [min]**	**Molecular weight [g/mol]**	**Δm**	**reaction**	**product percentage *in vitro* [%]**
**Testosterone 1**	12.0	288.4	0		/
2β-Hydroxytestosterone **2**	10.9	304.4	+16	hydroxylation at C-2 in β-position	70
4-Androstene-3,17-dione **3**	11.6	286.4	-2	oxidation to ketone at C-17	3
Hydroxylation product **4**	10.6	304.4	+16	hydroxylation at unknown position	12
Hydroxylation product **5**	9.7	304.4	+16	hydroxylation at unknown position	2
Hydroxylation product **6**	9.8	304.4	+16	hydroxylation at unknown position	1
Oxidation product **7**	11.5	286.4	-2	proposed: oxidation to ketone	1
Double oxidation product **8**	7.1	320.4	+32	double hydroxylation	3
Double oxidation product **9**	9.4	320.4	+32	double hydroxylation	5
Double oxidation product **10**	10.1	320.4	+32	double hydroxylation	3

Supplementary Results

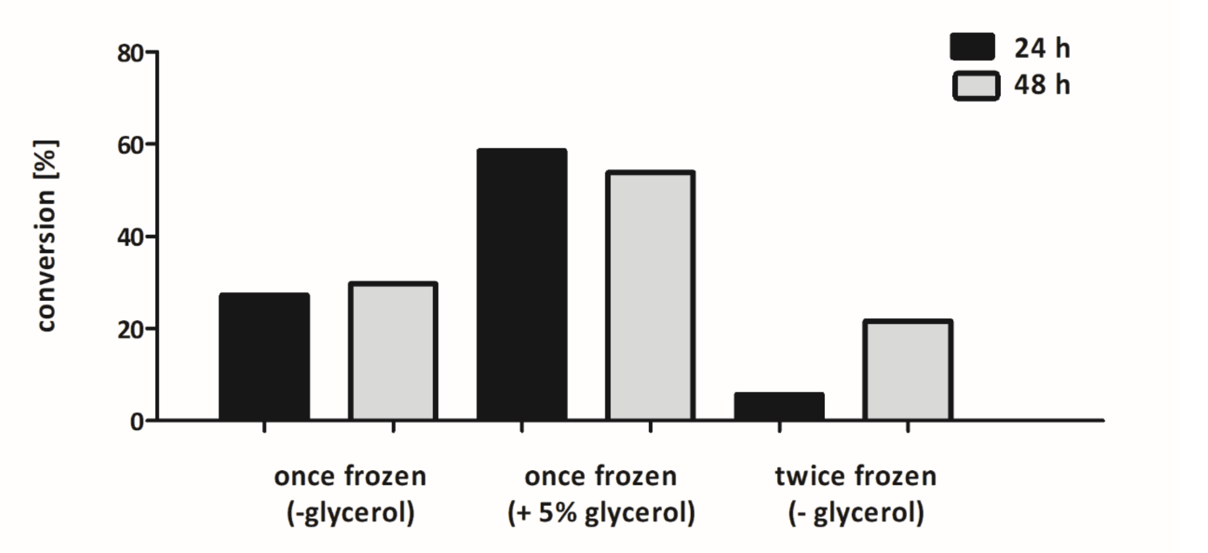

Figure S1: Effect of freezing and glycerol addition during lyophilization on conversion catalyzed by *E. coli* C43 (DE3) pET22b-*cyp105D* + pCOLADuet- *pdx-pdr-adh. E. coli* cells were once or twice frozen at -80°C and then lyophilized for either 24 h (black) or 48 h (grey). Reaction conditions: 1 mM testosterone in 5 % (v/v) propan-2-ol final concentration, 25 °C, 1100 rpm shaking frequency, 0.5 mL reaction volume in 2 mL tubes, 10 mg/mL lyophilized cells, reaction time 20 h. The biotransformation was performed in technical duplicates. For simplicity, the product percentage is not stated.

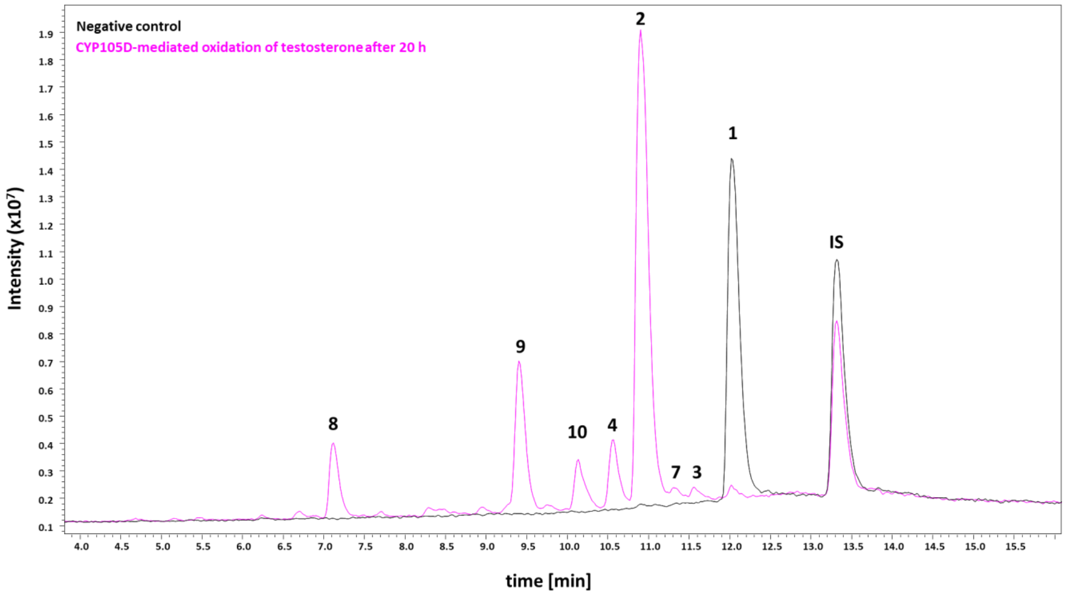

Figure S2: Exemplary LC/MS-chromatogram showing the oxidation of testosterone 1 to the products 2-10 by the CYP105D-based *E. coli* whole-cell biocatalyst (pink) in comparison to a negative control (black). The characteristics of the products are provided in Table S3. Products 5 and 6 were not detected in this experiment. IS: internal standard progesterone.

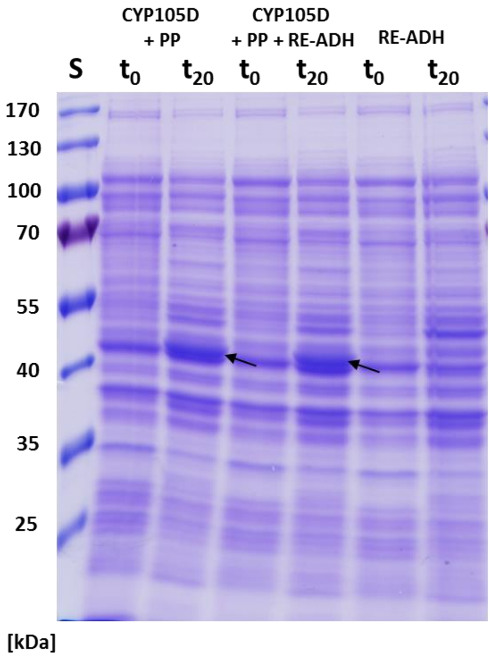

Figure S3: SDS-PAGE analysis of *E. coli* C43 (DE3) strains for whole-cell biocatalysis. The picture shows the comparison of whole-cell samples before induction (t_0) and 20 h after induction (t_{20}) as follows: *E. coli* C43 (DE3) pET22b-*cyp105D* + pCOLADuet-PP (lane 1 and 2), *E. coli* C43 (DE3) pET22b-*cyp105D* + pCOLADuet-PPRE (lane 3 and 4), *E. coli* C43 (DE3) pCOLADuet-Re-ADH (lane 5 and 6), PageRuler prestained protein ladder (lane S). CYP105D (43.9 kDa) is marked with a black arrow. Pdx (11.5 kDa), Pdr (45.8 kDa) and RE-ADH (36.2 kDa) were not detected.

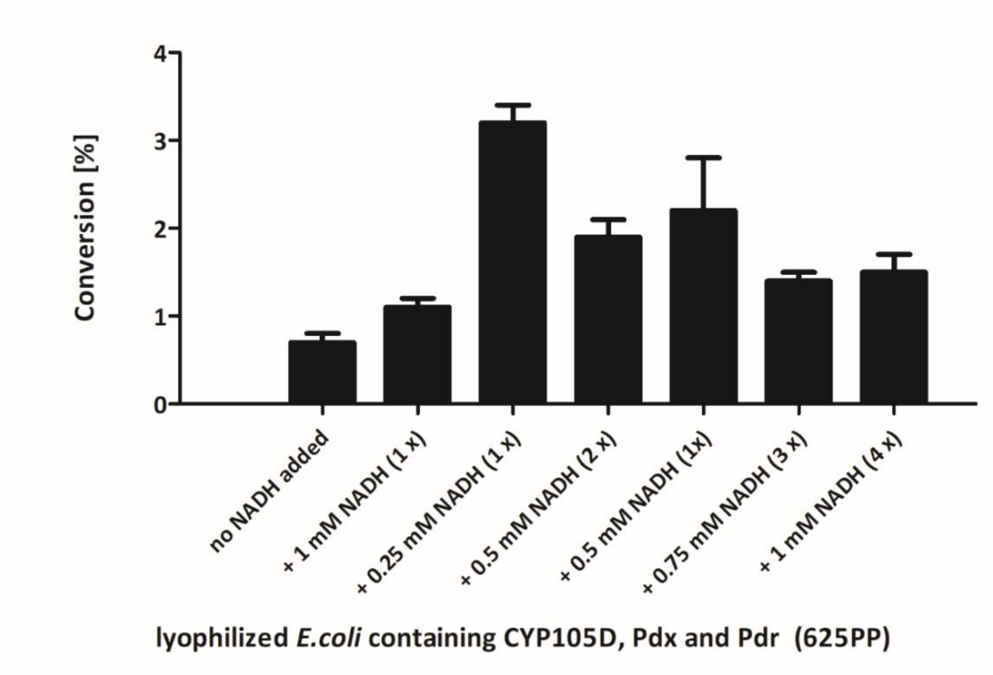

Figure S4: Effect of NADH addition on testosterone 1 conversion mediated by the lyophilized whole-cell catalyst without ADH. NADH was added up to four times (number in brackets) every 2 h. Reaction conditions: 1 mM testosterone in 5 % (v/v) propan-2-ol final concentration, 25 °C, 1100 rpm shaking frequency, 0.5 mL reaction volume in 2 mL tubes, 10 mg/mL lyophilized cells, reaction time 20 h. 0.25 mM NADH was added up to four times at 0 h, 2 h, 4 h and 6 h incubation. The biotransformation was performed in technical duplicates. Because of the overall low conversions, the product percentage is not stated.

References

Abokitse, K., Hummel, W., 2003. Cloning, sequence analysis, and heterologous expression of the gene encoding a (*S*)-specific alcohol dehydrogenase from *Rhodococcus erythropolis* DSM 43297. Appl. Microbiol. Biotechnol. 62, 380-386.

Hilberath, T., Windeln, L.M., Decembrino, D., Le-Huu, P., Bilsing, F.L., Urlacher, V.B., 2020. Two-step screening for identification of drug-metabolizing bacterial cytochromes P450 with diversified selectivity. ChemCatChem 12, 1710-1719.
